# Health technology assessment and price negotiation alignment for rare disorder drugs in Canada: Who benefits?

**DOI:** 10.1186/s13023-022-02390-x

**Published:** 2022-06-13

**Authors:** Nigel S. B. Rawson

**Affiliations:** 1 Saskatoon, SK Canada; 2 Canadian Health Policy Institute, Toronto, ON Canada; 3 Macdonald-Laurier Institute, Ottawa, ON Canada

**Keywords:** Rare diseases, Orphan drugs, Health technology assessment, Canada

## Abstract

**Background:**

Since 2014, the Canadian Agency for Drugs and Technologies in Health (CADTH), which performs health technology assessments for all federal, provincial and territorial government drug programs (except Quebec’s) and the pan-Canadian Pharmaceutical Alliance (pCPA), which conducts price negotiations with manufacturers for all government drug programs, have been aligning their processes.

**Objective:**

To examine trends in CADTH recommendations for non-oncology drugs for rare disorders (DRDs) released between 2014 and 2021, results of pCPA negotiations for the same drugs, and listings in government drug plans to assess who benefits from the alignment.

**Results:**

Recommendations were positive in 87% of the reviews, although all included clinical criteria for use and/or economic conditions. Almost 90% of the DRDs with a positive recommendation had a successful price negotiation and 71% of those with a negative recommendation had no negotiation. Although no recommendation published before mid-2016 had a specified price reduction, almost 95% of those issued afterwards included the price reduction required to achieve a specific low cost-effectiveness threshold. The median time between the DRDs receiving marketing approval and a completed price negotiation was 663 days. Negotiations for DRDs completed after 2017 generally had fewer listings in government drug plans, but there was no distinct trend. The drug’s price likely played a role in listing decisions. When DRDs were listed, drug plans had access criteria consistent with CADTH’s or stronger for all the DRDs.

**Conclusions:**

The governments who own, fund and manage CADTH and the pCPA benefit from their alignment. The alignment is less beneficial for patients waiting for access to the DRDs. The time taken by CADTH and pCPA actions and individual government drug plans to make listing decisions delays access. CADTH’s clinical criteria have become more extensive and are applied rigorously by drug plans which restricts patient access to DRDs. Canadians with rare disorders urgently need their governments to implement a long-overdue, comprehensive rare disease strategy to ensure DRDs are reviewed and reimbursed quickly and equitably to provide adequate health care to all who need them.

## Background

Universal government health care insurance covering physician, hospital (including medicines administered there) and laboratory services exists in Canada, but medicines dispensed in the community are not included. Federal, provincial and territorial governments have their own prescription drug plans offering a varying degree of coverage to about a quarter of the population comprising seniors, social assistance recipients, children and some other special groups, such as cancer patients, or when costs are deemed catastrophic.

Several obstacles must be overcome by pharmaceutical manufacturers to have a drug listed in government drug plans. The first is health technology assessment performed for all federal, provincial and territorial drug plans (except in Quebec) by the Canadian Agency for Drugs and Technologies in Health (CADTH) [1]. CADTH recommendations are commonly qualified with clinical criteria and/or a need for a price reduction. Following a positive recommendation, pharmaceutical manufacturers usually look to be invited into the federal, provincial (including Quebec) and territorial governments’ collective price negotiating process, known as the pan-Canadian Pharmaceutical Alliance (pCPA). If a price agreement is reached, a letter of intent (LOI) is signed that implies the drug will be listed in any subsequent agreement with drug plans with an established price and listing criteria [2]. The negotiation process and agreement terms are not publicly available.

Government drug plans are not mandated to reimburse a medicine that has been successfully negotiated with the pCPA. Using the pCPA’s LOI terms, manufacturers negotiate individual product listing agreements with each participating jurisdiction. Individual negotiations with drug plans are confidential so that, when a medicine is not listed, it is unknown whether this is due to its government opting out of the pCPA negotiation or opting in but failing to reach its own listing agreement with the manufacturer.

A previous evaluation of drugs for rare disorders (DRDs) with CADTH recommendations issued between January 2004 and February 2016 found that the positive recommendation rate decreased as the prevalence of the DRD’s indication decreased, while an increase occurred in reports in which CADTH recommended a price reduction [3]. Before 2012, high cost was a factor in 85% of negative recommendations, whereas between 2012 and February 2016, no DRD with a negative recommendation had its cost noted in the reimbursement report and 100% of those with a positive recommendation included the need for a price reduction.

CADTH and the pCPA have continued to align their processes. Since insufficient recommendations were available in the post-2015 period in the previous work to fully assess the impact of the alignment on the outcomes of price negotiations and listing in government drug plans, the objective of this analysis was to examine trends in reimbursement recommendations for non-oncology DRDs published between 2014 and 2021, results of pCPA negotiations for the same drugs, and listings in government drug plans. Oncology drugs were excluded because their coverage is dealt with differently in most provinces.

## Methods

Non-oncology DRDs with final recommendations published between January 2014 and December 2021 were identified from CADTH’s reimbursement review webpage [1]. Only DRDs for indications with a prevalence of ≤ 10 per 100,000 population were included to ensure that drugs for unquestionably rare disorders were the focus. Prevalence was obtained from the Orphanet website [4] or up-to-date publications when Orphanet provided a wide range or no data.

Information recorded from the CADTH reimbursement review reports included the drug, its indication, dates of submission to CADTH and final recommendation, the recommendation, any estimate of the daily cost of treatment, and whether reviewers expressed concern about the drug’s efficacy or cost-effectiveness. The pCPA website [5] was searched for corresponding negotiations for the same medicines. Dates on which negotiations were begun and completed or declined were recorded, together with the outcomes.

Formularies, special benefit lists and bulletins of each of the 10 provinces, together with the formulary of the federal Non-Insured Health Benefits plan for First Nations and Inuit people, as at the end of December 2021 were reviewed to assess how many of the DRDs were reimbursed in these programs.

Clinical criteria for drug access specified in positive CADTH recommendations were categorized as minimal, moderate or extensive. Minimal criteria simply stated what type of patients are to receive the drug and that they should be under the care of an appropriate specialist. Moderate criteria went beyond this by adding one or more diagnostic tests commonly used in patients with the condition for which the drug is indicated. Extensive criteria broadened the requirements to include one or more specialized diagnostic tests, such as those for specific gene mutations. Drug plan access criteria were assessed as being the same, weaker (less restrictive) or stronger (more restrictive) when compared with CADTH’s criteria.

## Results

Fifty-four CADTH final recommendations published between January 2014 and December 2021 for 47 unique non-oncology DRDs were identified (Table 1). Most DRDs (42; 77.8%) were indicated for a genetic disorder. The times taken for CADTH’s reviews had a median of 215 days (inter-quartile range: 188–272 days). No evidence was seen in the CADTH reports of any significant delay caused by a request to the manufacturer for additional data.

**Table 1:** Outcomes of 54 reimbursement reviews and price negotiations for rare disorder drugs, 2014–2021

Generic (brand) names	Clinical indication	CADTH reimbursement review			pCPA price negotiation outcome
		Completed	Outcome	Price reduction	
Riociguat (Adempas)	Pulmonary thromboembolic hypertension	7/2014	LwC	Substantial reduction	LOI
Stiripentol (Diacomit)	Dravet syndrome^a^	10/2014	LwC	Reduction	LOI
Icatibant (Firazyr)	Hereditary angioedema^a^	12/2014	LwC	Reduction	LOI
Ivacaftor (Kalydeco)	Cystic fibrosis gating mutations^a^	12/2014	LwC	Substantial reduction	Closed, no LOI
Macitentan (Opsumit)	Pulmonary arterial hypertension	1/2015	LwC	Reduction	LOI^c^
Pasireotide (Signifor)	Cushing's disease	2/2015	DNR	Lack of C-E evidence	No negotiation
Elosulfase alfa (Vimizim)	Mucopolysaccharidosis IVA^a^	3/2015	DNR	Not C-E	No negotiation
Pirfenidone (Esbriet)	Idiopathic pulmonary fibrosis	4/2015^b^	LwC	Substantial reduction	LOI
Lomitapide (Juxtapid)	Homozygous familial hypercholesterolemia^a^	4/2015	DNR	Not C-E	No negotiation
Nintedanib (Ofev)	Idiopathic pulmonary fibrosis	10/2015	LwC	Substantial reduction	LOI
Taliglucerase alfa (Elelyso)	Gaucher disease^a^	10/2015	DNR	C-E not assessable	LOI
Ivacaftor (Kalydeco)	Cystic fibrosis R117H gating mutation^a^	11/2015	LwC	Substantial reduction	LOI
Riociguat (Adempas)	Pulmonary arterial hypertension	12/2015	LwC	Substantial reduction	Closed, no LOI
Galsulfase (Naglazyme)	Mucopolysaccharidosis VI^a^	2/2016	LwC	Substantial reduction	No negotiation
Asfotase alfa (Strensiq)	Hypophosphatasia^a^	3/2016	LwC	Substantial reduction	LOI^d^
Sodium phenylbutyrate (Pheburane)	Urea cycle disorders^a^	4/2016	LwC	Lack of C-E evidence	LOI
Elosulfase alfa (Vimizim)	Mucopolysaccharidosis IVA^a^	5/2016^b^	LwC	Substantial reduction	LOI
Teduglutide (Revestive)	Short bowel syndrome, adult	7/2016	LwC	> 80% for $50,000/QALY	LOI
Sapropterin (Kuvan)	Phenylketonuria^a^	10/2016^b^	LwC	> 90% for $50,000/QALY	LOI
Lumacaftor/ivacaftor (Orkambi)	Cystic fibrosis, F508del mutation^a^	10/2016	DNR	98% for $50,000/QALY	No negotiation
Selexipag (Uptravi)	Pulmonary arterial hypertension	10/2016	LwC	> 42% for $50,000/QALY	LOI
Glycerol phenylbutyrate (Ravicti)	Urea cycle disorders^a^	3/2017	LwC	Not C-E at $50,000/QALY	LOI
Obeticholic acid (Ocaliva)	Primary biliary cholangitis	7/2017	LwC	> 60% for $50,000/QALY	LOI
Eliglustat (Cerdelga)	Gaucher disease^a^	7/2017	LwC	Not to exceed similar drugs	Closed, no LOI
Nusinersen (Spinraza)	Spinal muscular atrophy^a^	12/2017^b^	LwC	95% for > $400,000/QALY	LOI
Migalastat (Galafold)	Fabry disease^a^	1/2018	LwC	Lower than similar drugs	LOI
Cysteamine (Procysbi)	Nephropathic cystinosis^a^	1/2018	LwC	> 95% for $100,000/QALY	LOI
Nitisinone (Orfadin)	Tyrosinemia type 1^a^	2/2018	LwC	> 87% for $50,000/QALY	LOI
Tocilizumab (Actemra)	Giant cell arteritis	3/2018	LwC	68% for $50,000/QALY	LOI
Nitisinone (MDK-Nitisinone)	Tyrosinemia type 1^a^	4/2018	LwC	> 87% for $50,000/QALY	LOI
Nitisinone (Nitisinone)	Tyrosinemia type 1^a^	8/2018	LwC	> 87% for $50,000/QALY	LOI
Lumacaftor/ivacaftor (Orkambi)	Cystic fibrosis, F508del mutation^a^	9/2018^b^	DNR	97% for $100,000/QALY	LOI^d^
Sebelipase alfa (Kanuma)	Lysosomal acid lipase deficiency^a^	9/2018	LwC	> 97% for $50,000/QALY	LOI
Nusinersen (Spinraza)	Spinal muscular atrophy^a^	2/2019	LwC	Not C-E at $300,000/QALY	LOI
Edaravone (Radicava)	Amyotrophic lateral sclerosis	3/2019	LwC	> 95% for $200,000/QALY	LOI
Cerliponase alfa (Brineura)	Neuronal ceroid lipofuscinosis type 2^a^	5/2019	LwC	> 99% for $50,000/QALY	LOI
Mercaptamine (Cystadrops)	Cystinosis^a^	6/2019	LwC	> 97% for $50,000/QALY	LOI
Patisiran (Onpattro)	Transthyretin amyloidosis^a^	7/2019	LwC	98% for $50,000/QALY	LOI
Lanadelumab (Takhzyro)	Hereditary angioedema^a^	11/2019	LwC	> 58% for $50,000/QALY	LOI
Teduglutide (Revestive)	Short bowel syndrome, pediatric	11/2019	LwC	> 80% for $50,000/QALY	LOI
Inotersen (Tegsedi)	Transthyretin amyloidosis^a^	12/2019	LwC	> 88% for $50,000/QALY	LOI
Tafamidis (Vyndaqel)	Transthyretin amyloidosis^a^	2/2020	LwC	> 92% for $50,000/QALY	LOI
Burosumab (Crysvita)	Hypophosphatemia (X-linked)^a^	5/2020	LwC	94% for $50,000/QALY	LOI
Eculizumab (Soliris)	Neuromyelitis optica spectrum disorder^a^	8/2020	LwC	96% for $50,000/QALY	Active
Caplacizumab (Cablivi)	Thrombotic thrombocytopenic purpura^a^	8/2020	DNR	75% for $50,000/QALY	No negotiation
Voretigene neparvovec (Luxturna)	Leber’s congenital amaurosis^a^	11/2020	LwC	> 74% for $50,000/QALY	Active
Onasemnogene abeparvovec (Zolgensma)	Spinal muscular atrophy^a^	3/2021	LwC	> 90% for $50,000/QALY	LOI
Amifampridine (Ruzurgi)	Lambert-Eaton myasthenic syndrome^a^	4/2021	LwC	Not C-E at $50,000/QALY	Active
Satralizumab (Enspryng)	Neuromyelitis optica spectrum disorder^a^	4/2021	LwC	> 89% for $50,000/QALY	Active
Luspatercept (Reblozyl)	Beta-thalassemia associated anemia^a^	6/2021	LwC	> 85% for $50,000/QALY	Under consideration
Risdiplam (Evrysdi)	Spinal muscular atrophy^a^	8/2021	LwC	99% for > $50,000/QALY	Active
Elexacaftor/tezacaftor/ivacaftor (Trikafta)	Cystic fibrosis, F508del mutation^a^	9/2021	LwC	> 90% for $50,000/QALY	LOI
Givosiran (Givlaari)	Acute hepatic porphyria^a^	9/2021	LwC	> 57% for $50,000/QALY	Under consideration
Trientine (MAR-Trientine)	Wilson’s disease^a^	11/2021	LwC	27% for $50,000/QALY	Active

CADTH, Canadian Agency for Drugs and Technologies in Health; C-E, cost-effectiveness; DNR, do not reimburse; LOI, letter of intent; LwC, list with conditions; pCPA, pan-Canadian Pharmaceutical Alliance; QALY, quality-adjusted life-year

^a^Genetic disorder

^b^Resubmission to CADTH

^c^Earlier negotiation closed with no agreement

^d^Earlier pCPA decision not to negotiate

Recommendations were positive in 47 (87.0%) of the reviews; all included clinical criteria for use and/or economic conditions. In the seven reports with a negative recommendation, CADTH reviewers expressed concern about the DRD’s efficacy, despite having been assessed by Health Canada’s regulatory review as acceptable.

Table 1 also demonstrates that none of the 17 recommendations issued before mid-2016 had a specified price reduction, whereas almost all (35; 94.6%) of the 37 published afterwards included the price reduction required to achieve a specific incremental cost-effectiveness threshold. For 30 (81.1%), the threshold was $50,000 per quality-adjusted life-year (QALY). Since mid-2019, all recommendations used this low threshold.

For 46 of the CADTH recommendations, a corresponding pCPA decision was identified (Table 1). A price negotiation was active or the pCPA was considering whether to enter a negotiation for the other eight. The median time required for the pCPA negotiations was 224 days (inter-quartile range: 159–314 days).

Thirty-five (89.7%) of the 39 CADTH reviews with a positive recommendation had a pCPA negotiation resulting in a LOI. A price negotiation was undertaken but closed without a LOI for three of the remaining drugs, while the pCPA decided not to pursue a negotiation for the other. Negotiations were also not pursued for five DRDs with a negative recommendation; the other two had a successful negotiation, although that for lumacaftor/ivacaftor was only achieved after the pCPA had initially refused to negotiate.

Reimbursement reviews and price negotiations take a significant amount of time. The median of the time between the DRDs receiving marketing approval from Health Canada and either a pCPA completed negotiation (with or without a LOI) or a decision not to negotiate was 663 days (inter-quartile range: 425–1046 days).

Table 2 shows numbers of drug plans listing the 38 unique DRDs with completed pCPA negotiations as at the end of 2021. Although negotiations for DRDs completed after 2017 generally had fewer listings, there was no distinct trend. However, the DRD’s cost likely played a role in listing decisions based on daily list prices available from the CADTH reports. The median cost of DRDs listed in nine to 11 drug plans was $128 (inter-quartile range: $112–$263), whereas the median for DRDs listed in six to eight plans was $655 (inter-quartile range: $419–$918) and for those listed in fewer than six plans was $1619 (inter-quartile range: $752–$2633).

**Table 2:** Drug plan listings for 33 unique rare disorder drugs with successful price negotiations

Generic (brand) names	Clinical indication	Date most recent price negotiation completed	Drug plan listings^a^	
			No	%
Riociguat (Adempas)	Pulmonary hypertension	1/2015	9	81.8
Stiripentol (Diacomit)	Dravet syndrome	5/2015	10	90.9
Icatibant (Firazyr)	Hereditary angioedema	8/2015	11	100.0
Nintedanib (Ofev)	Idiopathic pulmonary fibrosis	8/2016	11	100.0
Pirfenidone (Esbriet)	Idiopathic pulmonary fibrosis	9/2016	11	100.0
Sodium phenylbutyrate (Pheburane)	Urea cycle disorders	11/2017	8	72.7
Selexipag (Uptravi)	Pulmonary arterial hypertension	12/2017	10	90.9
Glycerol phenylbutyrate (Ravicti)	Urea cycle disorders	12/2017	10	90.9
Asfotase alfa (Strensiq)	Hypophosphatasia	1/2018^b^	5	45.4
Taliglucerase alfa (Elelyso)	Gaucher disease	5/2018	4	36.4
Obeticholic acid (Ocaliva)	Primary biliary cholangitis	6/2018	11	100.0
Cysteamine (Procysbi)	Nephropathic cystinosis	7/2018	7	63.6
Migalastat (Galafold)	Fabry disease	8/2018	5	45.4
Nitisinone (Orfadin and generics)	Tyrosinemia type 1	11/2018	6	54.5
Elosulfase alfa (Vimizim)	Mucopolysaccharidosis IVA	11/2018^c^	3	27.3
Tocilizumab (Actemra)	Giant cell arteritis	12/2018	8	72.7
Nusinersen (Spinraza)	Spinal muscular atrophy	6/2019	7	63.6
Ivacaftor (Kalydeco)	Cystic fibrosis gating mutations	7/2019^b^	8	72.7
Mercaptamine (Cystadrops)	Cystinosis	8/2019	6	54.5
Cerliponase alfa (Brineura)	Neuronal ceroid lipofuscinosis type 2	1/2020	4	36.4
Sapropterin (Kuvan)	Phenylketonuria	2/2020	5	45.4
Edaravone (Radicava)	Amyotrophic lateral sclerosis	4/2020	10	90.9
Inotersen (Tegsedi)	Transthyretin amyloidosis	4/2020	7	63.6
Teduglutide (Revestive)	Short bowel syndrome	10/2020	7	63.6
Lanadelumab (Takhzyro)	Hereditary angioedema	10/2020	7	63.6
Sebelipase alfa (Kanuma)	Lysosomal acid lipase deficiency	10/2020	6	54.5
Patisiran (Onpattro)	Transthyretin amyloidosis	11/2020	8	72.7
Macitentan (Opsumit)	Pulmonary arterial hypertension	12/2020^c^	6	54.5
Tafamidis (Vyndaqel)	Transthyretin amyloidosis	2/2021	8	72.7
Lumacaftor/ivacaftor (Orkambi)	Cystic fibrosis, F508del mutation	6/2021^d^	6	54.5
Elexacaftor/tezacaftor/ ivacaftor (Trikafta)	Cystic fibrosis, F508del mutation	9/2021	9	81.8
Burosumab (Crysvita)	Hypophosphatemia (X-linked)	9/2021	3	27.3
Onasemnogene abeparvovec (Zolgensma)	Spinal muscular atrophy	10/2021	2	18.2

^a^At December 31, 2021

^b^Negotiation in 2017 closed with no agreement

^c^Negotiation in 2015 closed with no agreement

^d^Decision not to pursue negotiations in 2016 and 2019

**Fig. 1:**
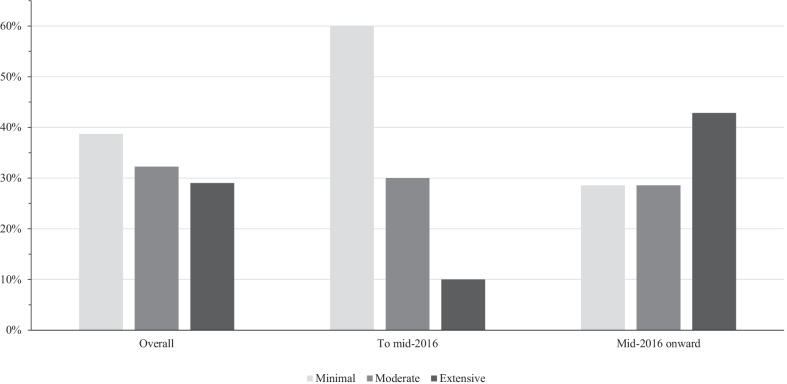
Canadian Agency for Drugs and Technologies in Health clinical criteria for rare disorder drugs

When CADTH clinical criteria for DRDs with positive recommendations were classified into minimal, moderate or extensive, DRDs with recommendations published after mid-2016 were more likely to have extensive criteria than those issued previously (Fig. 1). Government drug plans had access criteria consistent with CADTH’s criteria or stronger for all the DRDs; none had weaker criteria. A third or more of the listing drug plans had stronger access criteria for asfotase alfa for hypophosphatasia (two of the four plans with listing criteria accessible required additional tests and documented disease-related skeletal abnormalities) and elosulfase alfa for mucopolysaccharidosis IV (one of the two plans listing this DRD required additional orthopedic, respiratory, ophthalmologic and mobility assessments).

## Discussion

This evaluation indicates that the CADTH-pCPA alignment has strengthened in recent years. Unlike the earlier analysis [3], no evidence was found that drugs for ultra-rare disorders had a greater probability of receiving a negative recommendation from CADTH. The few DRDs that received a negative recommendation generally did so because reviewers had concerns about efficacy. Their developers were unlikely to be invited to negotiate with the pCPA.

The majority of CADTH reviews resulted in a positive recommendation with clinical access conditions and/or a price reduction. CADTH’s clinical criteria have become more extensive, which may in part be due to more DRDs for specific gene mutations being launched. Price criteria commonly included a specific percentage reduction to achieve a cost-effectiveness threshold of $50,000 per QALY, which has apparently become CADTH’s standard. Price reduction recommendations range widely but were at least a 75% reduction for three-quarters of them.

Price recommendations to achieve a low $50,000 per QALY threshold are not restricted to DRDs. Similar recommendations can be found in reviews of drugs for common disorders. In fact, CADTH reimbursement reports have recently been restructured to require a pricing statement, despite the agency’s role not being price regulation. CADTH’s price recommendations are clearly intended to establish a starting point for pCPA negotiations. Nevertheless, a successful price negotiation was achieved for almost 90% of the DRDs with a positive recommendation. It seems unlikely that manufacturers submitted to price cuts of 75% or more.

Who benefits from the alignment between CADTH and the pCPA?

The alignment works well for the federal, provincial and territorial governments who own, fund and manage CADTH and the pCPA, as well as their own drug programs [6]. Government drug plans participating in a successful pCPA negotiation are not mandated to cover the medication. The likelihood that a DRD will be listed by most plans increased as time elapsed after a successful price negotiation but decreased as their cost increased. Delaying access means not having to pay for new listings. It also means manufacturers are hindered in selling their products and have less time to benefit from patent protection, while patients have to wait longer for access to potentially beneficial medicines.

The time taken by CADTH and the pCPA to complete their work resulted in access being delayed by more than a year for three-quarters of the DRDs and nearly three years for 25%. This on top of the time Health Canada takes to review and approve the DRDs for marketing (median 258 days, inter-quartile range: 210–357 days). Additional delays are caused by individual government drug plans deciding whether to list the DRDs. The entire process between marketing approval and government drug plan listing can take several years. For one DRD, sapropterin (Kuvan), it took almost 10 years since marketing approval to obtain a positive CADTH recommendation and complete a successful pCPA price negotiation [7] and the drug is still only listed in five government plans with restrictive access criteria.

Dates on which government drug plans decided to list the DRDs were not available for this analysis. However, a recent assessment of 63 medicines with an orphan drug designation approved by the European Medicines Agency between January 2015 and March 2020 demonstrated that the shortest median durations from Health Canada approval to reimbursement were 17.3 months in British Columbia and 19.6 months in Quebec and Manitoba, whereas in Europe, the shortest median durations from regulatory approval to a decision on reimbursement were 3.2 months in Austria, 4.1 months in Germany and 6.0 months in Finland [8]. It remains to be seen whether the work recently begun on a process to align regulatory reviews and health technology assessments in Canada reduces the duration between marketing approval and drug plan listing.

The current analysis also showed that, over the last eight years, clinical access criteria specified by CADTH have become more extensive and are applied rigorously by drug plans, which denies access for patients who do not satisfy the criteria [9]. Furthermore, higher cost DRDs were listed more slowly by drug plans, which especially impacts Canadians with ultra-rare disorders because drugs for these disorders tend to be more expensive.

Since 2017, Canada’s federal government has attempted to introduce major revisions in the regulations of the tribunal whose role is to prevent time-limited drug patents from being abused by excessive prices that would have significantly altered the country’s pharmaceutical environment [10]. This led to considerable uncertainty during the last five years in the biopharmaceutical industry and anxiety among patients, especially those wanting access to new ground-breaking medicines that offer solutions to unmet or poorly met health needs. Legal decisions against the federal government have led to a scale-back in the changes, which will be limited to an adjustment in the tribunal’s international list price comparison. Some but not all drug developers may accept this revision. Only time will tell whether DRDs are impacted more or less than other drugs by this change.

The government also intends to implement a national strategy for access to DRDs, but the affordability of DRDs has been over-emphasized so far [11]. High-cost ground-breaking DRDs that fulfill unmet needs for patients and also reduce expensive hospitalizations and other health services and are life-changing for patients but are unaffordable for the average Canadian must be part of the initiative. An urgent need exists for the federal, provincial and territorial governments to implement a long-overdue, comprehensive rare disease strategy [8, 12] that includes ensuring that DRDs are reviewed and reimbursed quickly and equitably to provide adequate health care to all Canadians that need them.

## Conclusions

The CADTH-pCPA alignment is working for the governments that own these agencies, but patient access continues to be delayed and an uphill battle [13]. Patients with rare diseases, such as amyotrophic lateral sclerosis, do not have time to wait, while children with developmental genetic diseases need to began therapy as soon as possible to avoid irreversible physical or mental deterioration. The focus in Canada needs to change from raising barriers to ensuring that patients with unmet needs can access high-cost innovative medicines that alleviate suffering, prevent premature death and/or significantly improve their quality of life. This involves changing the antagonist approach to the biopharmaceutical industry that Canadian governments have held for decades to one of cooperative collaboration [14].

## Data Availability

Dataset analyzed is available from the author on reasonable request.

## Abbreviations

CADTH: Canadian Agency for Drugs and Technologies in Health
DRD: Drug for rare disorder
LOI: Letter of intent
pCPA: pan-Canadian Pharmaceutical Alliance
QALY: Quality-adjusted life-year
